# Effect of cowpea flour processing on the chemical properties and acceptability of a novel cowpea blended maize porridge

**DOI:** 10.1371/journal.pone.0200418

**Published:** 2018-07-10

**Authors:** Theresa N. Ngoma, Ulemu K. Chimimba, Agnes M. Mwangwela, Chrissie Thakwalakwa, Kenneth M. Maleta, Mark J. Manary, Indi Trehan

**Affiliations:** 1 Department of Food Science and Technology, Lilongwe University of Agriculture and Natural Resources, Lilongwe, Malawi; 2 School of Public Health and Family Medicine, University of Malawi College of Medicine, Blantyre, Malawi; 3 Department of Pediatrics, Washington University in St. Louis, St Louis, Missouri, United States of America; 4 Department of Paediatrics and Child Health, University of Malawi College of Medicine, Blantyre, Malawi; US Department of Agriculture, UNITED STATES

## Abstract

Childhood growth stunting is a pervasive problem in Malawi and is in large part due to low quality complementary foods and chronic gut inflammation. Introducing legumes such as cowpea (*Vigna unguiculata*) into the complementary diet has the potential to improve childhood growth by improving diet quality through improvements in macro- and micronutrients and also by reducing gut inflammation. However, cowpea is relatively underutilized in complementary feeding in Malawi due to its strong taste, long processing time, and high energy requirements for processing. Effective utilization of cowpea in complementary feeding requires processing which may affect chemical composition as well as sensory quality. The present study evaluated the effect of processing on the retention of zinc, crude fibre, and flavonoid in roasted, boiled, and dehulled cowpea flours, and assessed the acceptability of maize porridge (70%) enriched with one of the three cowpea flours (30%). Roasting, dehulling, and boiling did not have any effect on zinc content. Crude fibre content increased after processing by all methods. Processing had no effect on measurable flavonoids. Roasted, boiled, and dehulled cowpea blended maize porridges were acceptable to children with mean quantities of leftover food of less than 3g from the given 100g. Caregivers also rated the blended flours to be highly acceptable to them as well, with maize porridge blended with dehulled cowpea flour the most acceptable to both children and caregivers. These results demonstrate that cowpea flour, processed by any of these three different methods, could serve as a useful addition to maize porridge for complementary feeding of children in sub-Saharan Africa.

## Introduction

Malawi, a developing country in sub-Saharan Africa, is still battling the pervasive scourge of childhood malnutrition [1]. The problem is most pressing in rural areas where complementary feeding consists mainly of porridge made from maize flour only. The lack of potable water and other sanitary structures such as improved latrines also places rural children at a high risk of frequent enteric infections and the development of environmental enteric dysfunction (EED), a subclinical condition which compromises intestinal ability to absorb nutrients, leading to physical and cognitive stunting, poor response to oral vaccines, and diminished adult potential [2, 3]. Efforts to improve EED using probiotics [4] or antibiotics [5] have proven insufficient, as the inflammation seen histologically in EED is almost certainly much more multifactorial in aetiology. A growing body of evidence [6, 7] suggests that a diet enriched in legumes decreases systemic markers of inflammation, making the use of legumes as part of a supplementary diet an appealing strategy to combat EED and stunting in vulnerable children.

Cowpea *(Vigna unguiculata)* is a widely-grown legume crop in Malawi. It is grown throughout the country, and production is high in areas where other legumes such as common beans *(Phaseolus vulgaris L*.*)* do not grow well [8]. Cowpea immature pods and leaves are mostly utilized as vegetables with the dry seeds eaten as relish (stew) [9]. Cowpea seeds are rich in protein (18–25%) [10] and the fat content in cowpea is 1.4%-2.7% [11], with about 6% crude fibre [12]. Amino acids derived from cowpea are an effective complement to those obtained from cereals [13, 14]. Cowpea also has more calcium than meat and iron content equal to milk. The content of water-soluble vitamins (thiamine, riboflavin, niacin) is comparable to the levels found in fish and lean meat [15–18]. Cowpea protein isolates have good functional properties, including foaming and emulsifying activities in addition to solubility [19]. Cowpeas also have significant anti-inflammatory effects, mediated by specific phenolic profiles and antioxidant activity [20], which may be helpful in mitigating EED. Additionally, cowpea’s high content of crude and dietary fibre, similar to other grain legumes, has significant potential for enhancing digestibility and helping maintain a healthy gut microbiota [21, 22]. Despite the good nutritional profile of cowpeas and its great potential to ameliorate EED, it remains under-utilized in childhood feeding in Malawi, a country where about 37% of children under-five are stunted, 11% are underweight and 2.7% wasted [1]. While the nutritional value of cowpea and the traditional Malawian complementary food, maize, have been described previously [23, 24], this study assessed selected compositional values of cowpea flours after three different forms of processing and the acceptability of maize porridge blended with these cowpea flours to young children and their caregivers in the local context of Lirangwe, Blantyre District, Malawi.

## Materials and methods

### Choosing cowpea variety

The Sudan 1 variety of cowpea was chosen as it is one of the common varieties grown in rural Malawi. Cowpeas were obtained from the stocks maintained by the Department of Crop and Soil Sciences at the Lilongwe University of Agriculture and Natural Resources. The seeds were planted in November 2013 and harvested in April 2014. The cowpea grains were stored at room temperature before processing and no pesticides were used during storage.

### Preparation of cowpea and whole maize flour

#### Roasted cowpea flour

Cowpeas were sorted to remove rotten peas and any other foreign materials. Approximately 1 kg of cowpeas was then dry roasted at a time in an aluminium household frying pan using a 1000W electric hot plate as the heat source. The roasting was done for 50 minutes at 120°-130°C with constant stirring [25]. The peas were then milled using a hammer mill (EFS 2T) fitted with a standard sieve (sieve number 8). The flour was then kept in plastic air tight container and stored at room temperature prior to the acceptability study.

#### Boiled cowpea flour

Cowpeas were boiled in tap water at 100°C for 40 minutes from the time boiling started using a ratio of 1 kg of cowpeas to 5 L tap water. After boiling, the water was drained and the cowpeas were removed and sun dried for up to 48 hours on a reed mat before being milled into flour, as was done with the roasted flour [26].

#### Soaked, dehulled cowpea flour

Raw cowpeas were thoroughly rinsed in tap water before being soaked for 11 hours in tap water at room temperature (25°C). The soaked cowpeas were then removed from the water before being dehulled manually. The dehulled cowpeas were then boiled in water at 100°C for 20 minutes. The cowpeas were then dried in the sun before being milled and stored as above [27].

#### Whole maize flour

Whole grain maize flour was made from grains purchased locally. Defects were removed by hand sorting and the maize was milled using a hammer mill and stored as above.

### Determination of crude fibre in cowpea and cowpea flours

Crude fibre content was determined using the Association of Official Analytical Chemists method 920.39 [28]. Approximately 2 g of powdered raw cowpea, roasted cowpea, boiled cowpea, or dehulled cowpea flours were individually weighed and extracted for 60 minutes with petroleum ether in a Soxhlet extractor. The extracted flour was then transferred in a thimble to a 1 litre flask. Dilute sulphuric acid (200ml) was brought to a boil; the boiled acid was then transferred into the flask containing the flour and connected to a refluxing apparatus for 30 minutes with constant rotation to keep the flour from adhering to the sides of the flask and out of the acid. The flask was then removed and the contents were filtered through filter paper and washed with boiling water until the washings were acid free. This step was repeated using sodium hydroxide solution. The residue was transferred into a gooch crucible prepared with a thin compact layer of asbestos, and the residues were washed first with hot water, then 15 mL of ethanol, and finally three successive washings with petroleum ether. The crucible and contents were dried at 105°C for 3 hours, then cooled and weighed. Crucibles and contents were dried again at 105°C for 30 min. The contents in the crucible were incinerated in a muffle furnace at 550°C until all the carbonaceous matter was burnt. Lastly the crucible was cooled in a desiccator and weighed. The crude fibre percentage was calculated by subtracting the weight of the crucible with contents after ashing from the weight of the crucible with contents before ashing. Samples were tested in triplicate.

### Flavonoid determination in cowpea and cowpea flours

About 10 g of cowpea flour was weighed and mixed with 50 mL of acidified methanol prepared in a ratio of 79:20:1 of CH_3_OH, H_2_O, and HCl. The mixture was incubated for 72 h in darkness for auto extraction, filtered through Whatman paper Number 2 and absorbance of the clear supernatant measured spectrometrically (Jenway 6405, Jenway, Staffordshire, United Kingdom) at 300, 530, and 657 nm using acidified methanol as the blank. Concentrations of flavonoids were measured spectrometrically at 300 nm and expressed as Abs g.db-1 [29]. The concentration in part per million (ppm) was calculated using the formula: y = 0.1974 x−0.0004, where y was the absorption in grams per dry matter and x is the concentration in ppm. The concentration in ppm was then converted and reported in mg/100g. Samples were tested in triplicate.

### Determination of zinc in cowpea and cowpea flours

An atomic absorbance spectrophotometer (200 Series AA System, Agilent, Santa Clara, USA) was used to determine zinc content in the raw cowpea, roasted cowpea flour, boiled cowpea flour, and dehulled cowpea flour at a wavelength of 213.9 nm. Approximately 2 g of sample was weighed and ashed in a muffle furnace (Carbolite, AAF 11/7) at 500°C. After ashing, 10 mL of HCl was added to the sample and evaporated in a sand bath under a fume hood with caution not to bake the residue. An additional 20 mL of HCl was then added to dissolve the residue. The mixture was then filtered into a 100 mL flask washing the residue and the filter paper with water and the resulting solution was diluted to volume with distilled water. Samples were tested in triplicate. Final zinc content was calculated based on a calibration curve using standard concentrations of 0, 0.5 ppm, 1.0 ppm, 1.5 ppm, and 2.0 ppm.

### Preparation of cowpea fortified maize porridge

Cowpea and maize flour were mixed in a 30:70 ratio. A paste was then made in a pot by mixing 1250 mL of cold water with 300 g of the composite flour to make a cold slurry. Boiled water (3750 mL) was then gradually added to the cold slurry with constant stirring until a paste was formed. The pot was then placed on a charcoal burner and left to simmer for 30 minutes while covered. Salt (50 g) was added to improve taste. The porridge was stored in food warmers to keep warm throughout the serving time. Approximately 100 g of porridge was served to each child and their guardians in white disposable cups.

### Acceptability study

Acceptability was assessed by measuring the amount of test food consumed and likeability assessed with a 5-point hedonic scale ranking with facial expressions, where 1 represented “dislike very much” and 5 represented “like very much” [27]. The study enrolled children aged 6–35 months who did not have severe or moderate acute malnutrition. The guardians of the children were also enrolled and asked to complete a baseline survey questionnaire and to provide their opinion of the food. Guardians signed consent forms and the study received approval from the University of Malawi College of Medicine Research and Ethics Committee and from the Blantyre District Health Officer.

For the test feeding, 100 g of test porridge was given to each child and the amount of food consumed after 15 minutes calculated by subtracting the post-test weight from the pre-test weight of left-over food. Hedonic likeability was done for the caretaker and child with the caretaker rating their own liking and that of their child. The acceptability study took place in the morning between 8 am and 11 am; caregivers had been instructed to arrive at the study site before their children consumed any breakfast. The same subjects tested each of the three recipes on six different days, three at home and three in the clinic under direct observation. The caregiver’s acceptability rating was based on their perception of the child’s acceptability of the food both at home and in the clinic.

### Statistical analyses

Data were analysed using ANOVA and paired difference analyses to test significant differences between means, at *p* < 0.05 to represent statistical significance. Statistical calculations were performed with GraphPad Prism version 7.03.

## Results

### Baseline survey and acceptability of cowpea blended porridge

The baseline characteristics of the 115 acceptability subjects are provided in Table 1. Nearly 95% of the study children had already been introduced to complementary feeding with maize porridge. Over half of the children were still breastfeeding and only a small percentage of subjects had ever been treated for acute malnutrition. All children had already been introduced to either maize or maize-soy porridge.

**Table 1:** Baseline characteristics of study subjects (N = 115).

Characteristic	Number	Percentage
Both parents alive	108	93.9
Father working	45	39.1
Mother working	21	18.3
Still breastfeeding	61	53.0
Eats meals as part of communal feeding	72	62.1
Ever treated for acute malnutrition	5	4.3

An average of less than 3 g out of an initial 100 g of food remained after 15 minutes by children receiving porridge enriched with roasted, boiled, or dehulled cowpea flour (Table 2). Caregivers rated that they liked the test food very much and there were no differences between the recipes. No differences were observed when comparing any subgroup of children, including age, gender, and any of the factors listed in Table 1.

**Table 2:** Acceptability of cowpea blended maize porridge.

Composite flour type	Food consumed (g)^a^	Caregiver report of overall food acceptability score^b^
Maize + roasted cowpea flour	97.14 ± 0.40	4.67 ± 0.57
Maize + boiled cowpea flour	97.22 ± 0.39	4.57 ± 0.71
Maize + dehulled cowpea flour	97.90 ± 0.29	4.66 ± 0.90

^a^Composite porridge consumed in 15 minutes after initial provision of 100 g of flour.

^b^Based on 5-point hedonic scale; 1 = “dislike very much”; 5 = “like very much”.

### Moisture, zinc, crude fibre, and flavonoids

Moisture content among the different cowpea formulations varied between 6.7 and 11.4 g per 100 g (Table 3). Roasted cowpea flour had the lowest moisture content, while boiled cowpea flour had the highest.

**Table 3:** Chemical results for different cowpea preparations.

Flour type	Moisture (g / 100 g)	Zinc (mg / 100 g)	Crude fibre (g / 100 g)	Flavonoid (mg / 100 g)
Raw cowpea	9.60 ± 0.13^a^	5.96 ± 0.12^e^	2.10 ± 0.06^g^	3.09 ± 0.11^j^
Roasted cowpea flour	6.71 ± 0.17^b^	5.75 ± 0.22^e^	3.13 ± 0.25^h^	4.67 ± 0.09^j^
Boiled cowpea flour	11.36 ± 0.50^c^	6.01 ± 0.13^e^	4.37 ± 0.35^i^	4.07 ± 0.19^j^
Dehulled cowpea flour	8.75 ± 0.20^d^	4.96 ± 0.19^f^	3.79 ± 0.08^i^	3.53 ± 0.10^j^

^a-j^ Values with the same superscript in the same column are not significantly different (p > 0.05), whereas those with different superscripts are significantly different from each other (p < 0.05).

Roasting, boiling and dehulling did not have a significant effect on the zinc content of the flours, except for a small decrease in the dehulled cowpea flour formulation. Cowpea flour would be expected to contribute at least 1.47 mg zinc per 100 g of maize-cowpea blended flour.

Crude fibre content after processing ranged between 3.1 g and 4.4 g per 100 g of flour, each significantly more than the 2.1 g in raw cowpea flour. The three different processing methods also resulted in a significant increase in total measurable flavonoids.

## Discussion

### Effect of processing methods on flour acceptability

All caregivers reported that their children were fed either corn or corn-soy porridge as their primary complementary food. Informal discussions with the caregivers revealed that most had occasional access to cowpea and had used soup from the cowpea stew to flavor their child’s porridge in the past, possibly contributing to the acceptance of cowpea. The observation that the study population was already familiar with communal feeding may have had a bearing on children’s acceptability as the test feeding was also done at a central place.

Acceptability results agreed with the observation made by Olapade, et al. [30], who enriched Ogi (fermented cereal gruel typically made from maize, sorghum, or millet) with instant cowpea powder as a complementary food and concluded that up to 30% cowpea had no significant adverse effects on sensory qualities. Odedeji, et al. [31] also found that complementary food formulations containing cowpeas and common beans were significantly more liked than formulations containing finger millet and dried peas. The acceptability results here contribute to the feasibility of cowpea for use in complementary feeding. Since maize and cowpea are all locally available and common in rural communities where children are most affected by EED and stunting, advocating in the community for maize porridge fortification with cowpea would seem to be quite achievable. Since none of the processing methods had a negative impact on the acceptability of the cowpea fortified porridge, the most affordable and reliable method of cowpea flour preparation could be pursued in order to make this fortification most widely available for complementary feeding.

### Effect of processing on chemical composition

The moisture content in all the flours was found to be below the maximum moisture limit for safe storage of 15% [31]. This low moisture content inhibits microbial growth in the flours [32], thereby extending its shelf life. Even though the process of making dehulled cowpea flour involved soaking in water, the moisture content in the dehulled cowpea flour was not remarkably high, perhaps because dehulling and splitting of the seeds into halves enhanced drying due to the increased surface to volume ratio.

The slightly lower zinc content observed in dehulled cowpea flour may be a result of soaking, which would allow zinc to leach into the water, as observed previously by Pereira [33]. However, all the cowpea preparation methods would indeed provide a substantial amount of zinc in children’s diets, especially considering that the daily recommended intake for children aged 1–3 years is 3 mg [24].

The observed increase in crude fibre following preparation is consistent with prior studies [34, 35], which also found an increase in crude fibre following soaking and cooking. The increase could be attributed to the formation of protein-fibre complexes [11] formed after chemical modifications induced by the soaking and cooking [35]. Though fibre is concentrated in the seed coat, the increase observed in dehulled flour may be a result of the longer soaking time (24 hours) and subsequent boiling. Crude fibre retained in the flours would enhance digestibility and help maintain a healthy gut microbiota, thereby improving overall gut health. Dietary fibre content of the final samples was subsequently evaluated in the United States after the larger clinical trial had begun [36].

The higher value of measurable flavonoids in roasted cowpea flour may be due to cell wall rupture and the breaking of bonds between antioxidant substances and the tissue matrix [37]. Heat causes disruption of cell membranes, which releases bound phytochemical compounds such as flavonoids into the medium, making them readily extractable [38].

## Conclusions

We have shown that a novel complementary food made by fortification of whole maize flour with three different forms of processed cowpea flours are acceptable by children and their caregivers in rural Malawi. These processing methods also produced flours that retained low moisture content and stable-to-improved zinc, crude fibre, and flavonoid content. Cowpea flour is thus a potential complementary food intervention that could be used towards mitigating stunting and EED in this high-risk population, especially since cowpea is available in Malawi and not entirely unfamiliar to the population. Given the relative simplicity in preparing the roasted cowpea flour and its equivalent nutritional content and acceptability, this novel formulation of complementary food was tested in two prospective, randomized clinical trials among four populations of rural Malawian children [39].

## Supporting information

S1 File Clinical information. Baseline clinical and demographic information for subjects enrolled in the acceptability trial.
(XLSX)

S2 File Clinical and demographic survey in Chichewa. (DOCX)

S3 File Acceptability measurement tool for home feeding in Chichewa. (DOCX)

S4 File Acceptability measurement tool for observed clinic feeding in Chichewa. (DOCX)

S5 File Clinical and demographic survey in English. (DOCX)

S6 File Acceptability measurement tool for home feeding in English. (DOCX)

S7 File Acceptability measurement tool for observed clinic feeding in English. (DOCX)

S8 File Caregiver acceptability measurement tool in Chichewa. (DOCX)

S9 File Caregiver acceptability measurement tool in English. (DOCX)
