# Supplementary material for: Effect of cowpea flour processing on the chemical properties and acceptability of a novel cowpea blended maize porridge
Source: PLoS One. 2018 Jul 10;13(7):e0200418. doi: 10.1371/journal.pone.0200418 (PMC6039016; doi:10.1371/journal.pone.0200418)
Supplement: S2 File — (DOCX) [file pone.0200418.s002.docx]

Mafunso oyambirira

**Sukulu ya ukachenjede ya LUANAR**

Nthambi yaukadaulo wa chakudya ndi luso

**Kafukufuku wa kalandieidwe ka phala lomwe mwaikidwa ufa wa nkhobwe**

Nambala yakafukufuku:______________________ Tsiku:_____________________________ Tsiku/ Mwezi / Chaka

Dzina lamwana: _______________________________________________

Usinkhu (miyezi): _____________________ **mwamuna / mkazi**

**Zaubale, chuma ndi zina**

1. Dzina lawosamalira mwana woyamba _________________________________________
2. Ubale pakati pawosamalra mwana ndi mwana:

**amayi / abambo / mchemwali / azakhali / agogo / ena _________**

1. Kodi amayi amwana ali moyo? **eya / ayi**
2. Kodi abambo amwana ali moyo? **eya / ayi**
   1. **Ngati eya→**kodi bambowo ali kunyumba? **eya / ayi**
3. Kodi bambowo amagwira ntchito kupatula kulima? **eya / ayi**
4. Kodi mayiwo amagwira ntchito kupatula kulima? **eya / ayi**
5. Kodi mwanayu ali ndi abale angati? **0 / 1 / 2 / 3 / 4 / 5 / 6 kapena ambiri**

**Mafunso ena** – aliyense ayankhe funso lililonse palokhapalokha komanso yekhayekha

1. Kodi mwanayu adakayamwabe? **eya / ayi**
   1. Ngati yankho ndi ayi**→ adaleka kuyamwa ali ndi miyezi ingati**? ________
   2. Ngati yankho ndi eya **→kodi mwanayu amayamwa kangati patsiku**? **0 / 1 / 2 / 3 / 4 / 5 / 6 kapena kambiri**
2. Kodi mwanayu adayamba kudya zakudya zina zoonjezera monga phala ali ndi miyezi ingati?____________
3. Ndi phala lanji lomwe amadya: **la chimanga / lamawere / lasoya / logula / lina**
4. Ndi zina ziti zoonjezera zomwe mumaziika mu phalalo: **shuga / mchere / zokometsera / zina ______**
5. Ndi ndani yemwe amamudyetsa mwanayu: **amayi / abambo / agogo / m’bale/ yekha / ena _______**
6. Kodi mwanayu amadya bwino pamalo ati? **pagulu / payekha**
7. Mumamudyetsa motani mwanayu, kugwiritsa ntchito … manja a mayi **/ manja a iye mwini/ ndi supuni / ndi kapu**
8. Kodi mwanayu anayamba waonetsapo zizindikiro zosonyeza kuti thupi lake limadana ndi nkhobwe monga zilonda (msungu kapena kusegula mmimba )? **eya / ayi**

1. Ndi masiku angati mmasiku asanu ndi awiri apitawa pamene mwanayu anadwala? **0 / 1 / 2 / 3 / 4 / 5 / 6 / 7**
   1. Anadwala matenda anji?___________________
2. Kodi mwanayu wamwa mankhwala mmasiku asanu ndi aiwiri apitawa? **eya / ayi**
   1. Ngati mwanayu wamwa mankhwala, chonde lembani mankhwalawo: ____________________________________
3. Kodi mwanayu wakhalapo ku NRU mmasiku asanu ndi awiri apitawa? **eya / ayi**
   1. Pazolinga zanji?_____________________
4. Kodi mwanayu anayamba walandirapo chithandizo chifukwa chosowa zakudya mthupi? **eya / ayi**
   1. Ngati ndi chomcho, ndi liti zinachitika izi? ______________________

Zinachitika kwanthawi yaitali bwanji?_______________________
