# Supplementary material for: Effect of cowpea flour processing on the chemical properties and acceptability of a novel cowpea blended maize porridge
Source: PLoS One. 2018 Jul 10;13(7):e0200418. doi: 10.1371/journal.pone.0200418 (PMC6039016; doi:10.1371/journal.pone.0200418)
Supplement: S3 File — (DOCX) [file pone.0200418.s003.docx]

Pepala losonyeza kudyera pakhomo

**Kafukufuku wa kalandilidwe ka phala lomwe mwaikidwa ufa wa khobwe**

**KUDYERA PAKHOMO**

Nambala yakafukufuku:______________________ Tsiku:_____________________________ Tsiku/ Mwezi / Chaka

Dzina lamwana: _______________________________________________

Usinkhu (miyezi): _____________________ **mwamuna / mkazi**

1. Kodi mwana akuwoneka motani: **wosinza watcheru ndi wodekha**

**wachidwi wonyatsidwa ndipo akilira**

1. kudya
   1. kuchuluka kwa chakudya poyamba kudya (mbale): **yozaza / theka / pang’ono / palibe**
   2. kuchuluka kwa chakudya chotsala (mbale): **yozaza / theka / pang’ono / palibe**
2. Kusatira zomwe zachitikazi, kodi mayi akuganiza kuti mwana wakonda phalali motani?

[
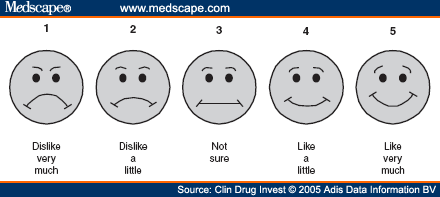
](https://www.google.com/url?sa=i&rct=j&q=&esrc=s&source=images&cd=&ved=0ahUKEwieiJXPtbbMAhWGvxQKHWiBCXUQjRwIBw&url=http://www.medscape.com/viewarticle/504566_2&psig=AFQjCNGdsoLJwwRWX7A0xEGvQXUzooUPjw&ust=1462107547486368)

1. Mongoyerekeza, ndi masupuni angati ozaza ndi phala omwe mwana wataya? **0 / 1 / 2 / 3 / 4 / 5 kapena ambiri**
2. Kodi mwanayu amafunika kumunyengerera kapena kumuthandiza kuti adye phalali? **eya/ ayi**
3. Kodi mwanayu anafuna phala lina loonjezera? **eya / ayi**
4. Zooneka pakafukufukuyu ___________________________________________________________________________________

___________________________________________________________________________________
