# Supplementary material for: Effect of cowpea flour processing on the chemical properties and acceptability of a novel cowpea blended maize porridge
Source: PLoS One. 2018 Jul 10;13(7):e0200418. doi: 10.1371/journal.pone.0200418 (PMC6039016; doi:10.1371/journal.pone.0200418)
Supplement: S4 File — (DOCX) [file pone.0200418.s004.docx]

Pepala losonyeza kadyedwe koyang’aniridwa

**Kafukufuku wa kalandieidwe ka phala lomwe mwaikidwa ufa wa nkhobwe**

Nambala yakafukufuku:______________________ Tsiku:_____________________________ Tsiku/ Mwezi / Chaka

Dzina lamwana: _______________________________________________

Usinkhu (miyezi): _____________________ **mwamuna / mkazi**

1. Kodi mwana akuwoneka motani: **wosinza watcheru ndi wodekha**

**wachidwi wonyatsidwa ndipo akilira**

1. Ndi nthawi iti yomwe mwana wadya yoyandikira tsopanoWhen was the child last fed (time): dzulo **/ mamawa wathawu / masanawa**

1. a. nthawi yoyambira kudya b. nthawi yotsirizira kudya

|___|___| |___|___| |___|___| |___|___|

maola mphindi maola mphindi

1. Kuchuluka kwachakudya chomwe wadya:
2. Kusatira zomwe zachitikazi, kodi mayi akuganiza kuti mwana wakonda phalali motani?

[
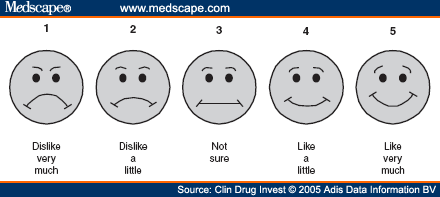
](https://www.google.com/url?sa=i&rct=j&q=&esrc=s&source=images&cd=&ved=0ahUKEwieiJXPtbbMAhWGvxQKHWiBCXUQjRwIBw&url=http://www.medscape.com/viewarticle/504566_2&psig=AFQjCNGdsoLJwwRWX7A0xEGvQXUzooUPjw&ust=1462107547486368)

1. Mongoyerekeza, ndi masupuni angati ozaza ndi phala omwe mwana wataya? **0 / 1 / 2 / 3 / 4 / 5 kapena ambiri**
2. Kodi mwanayu amafunika kumunyengerera kapena kumuthandiza kuti adye phalali? **eya/ ayi**
3. Kodi mwanayu anafuna phala lina loonjezera? **eya / ayi**
4. Zooneka pakafukufukuyu ___________________________________________________________________________________

___________________________________________________________________________________
