# Supplementary material for: Effect of cowpea flour processing on the chemical properties and acceptability of a novel cowpea blended maize porridge
Source: PLoS One. 2018 Jul 10;13(7):e0200418. doi: 10.1371/journal.pone.0200418 (PMC6039016; doi:10.1371/journal.pone.0200418)
Supplement: S5 File — (DOCX) [file pone.0200418.s005.docx]

Background information questionnaire

**L**ilongwe **U**niversity of **A**griculture and **N**atural **R**esources (LUANAR)

Department of Food Science and Technology

**COWPEA FORTIFIED PORRIDGE ACCEPTABILITY STUDY**

**BACKGROUND INFORMATION**

STUDY ID:______________________ DATE:_____________________________ Day / Month / Year

CHILD’S NAME: _______________________________________________

AGE (mos): _____________________ GENDER: **male / female**

**Social, Economic and Demographic Information**

1. Primary caregiver’s name _________________________________________
2. Primary caregiver’s relationship to child:

**Mother / Father / Sister / Aunt / Grandmother / Other _________**

1. Is the mother alive? **Yes / No**
2. Is the father alive? **Yes / No**
   1. **If YES →**Is the father in the home? **Yes / No**
3. Does the father have a job other than farming? **Yes / No**
4. Does the mother have a job other than farming? **Yes / No**
5. How many siblings does the child have? **0 / 1 / 2 / 3 / 4 / 5 / 6 or more**

**General Information** – Answer each question individually

1. Is the child still being breastfed? **Yes / No**
   1. If **NO→**At what age (in months) did the child STOP breastfeeding? ________
   2. If **YES →**How many times per day does the child breastfeed? **0 / 1 / 2 / 3 / 4 / 5 / 6 or more**
2. At what age (in months) did the child start eating complementary foods such as porridge?____________
3. What type of porridge is the child feeding on: **maize / wheat / soya / store-bought / other**
4. What other ingredients do you add to the child’s porridge: **sugar / salt / spices / other ______**
5. Who feeds the child: **mother / father / grandparent / sibling / child feeds self / other _______**
6. Under which setting does the child feed best? **Communal / isolated**
7. How do you feed the child, using… h**ands (mom’s) / hands (baby’s) / spoon / cup**
8. Has the child ever had symptoms of cowpea allergy (eg. Rash, diarrhea after eating)? **Yes / No**

1. How many days in the past 7 days has the child been otherwise ill? **0 / 1 / 2 / 3 / 4 / 5 / 6 / 7**
   1. What was the illness?___________________
2. Has the child taken any medications in the last 7 days? **Yes / No**
   1. If the child has taken medications, please list them: ____________________________________
3. Has the child been to the NRU in the past 7 days? **Yes / No**
   1. For what purpose?_____________________
4. Has the child ever been treated for malnutrition? **Yes / No**
   1. If so when? ______________________

For how long?_______________________
