# Supplementary material for: Effect of cowpea flour processing on the chemical properties and acceptability of a novel cowpea blended maize porridge
Source: PLoS One. 2018 Jul 10;13(7):e0200418. doi: 10.1371/journal.pone.0200418 (PMC6039016; doi:10.1371/journal.pone.0200418)
Supplement: S6 File — (DOCX) [file pone.0200418.s006.docx]

At-home feeding form

**COWPEA FORTIFIED PORRIDGE ACCEPTABILITY STUDY**

**AT-HOME FEEDING**

STUDY ID:_________________________ DATE:_____________________________ Day / Month / Year

AGE (mos): _____________________ GENDER: **male / female**

1. What is the child’s mood before eating? drowsy alert and calm

fussy irritable and crying

1. Consumption
   1. Amount of food at start of feeding (bowl): **full / half / little / none**
   2. Amount of leftover food (bowl): **full / half / little / none**
2. Based on the following scale, how much does the mother think the child likes the fortified porridge?

[
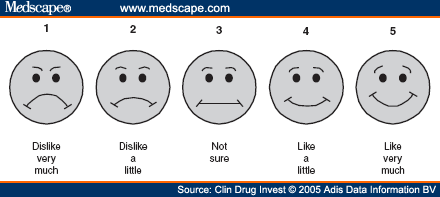
](https://www.google.com/url?sa=i&rct=j&q=&esrc=s&source=images&cd=&ved=0ahUKEwieiJXPtbbMAhWGvxQKHWiBCXUQjRwIBw&url=http://www.medscape.com/viewarticle/504566_2&psig=AFQjCNGdsoLJwwRWX7A0xEGvQXUzooUPjw&ust=1462107547486368)

1. Approximately how much food was spilled by child (spoonfuls)? **0 / 1 / 2 / 3 / 4 / 5 or more**
2. Did the child need coaxing or support to eat the study food? **Yes / No**
3. Did the child ask for more study food to eat? **Yes / No**
4. General observations ___________________________________________________________________________________

**____________________________________________________________________________________**
