# Supplementary material for: Effect of cowpea flour processing on the chemical properties and acceptability of a novel cowpea blended maize porridge
Source: PLoS One. 2018 Jul 10;13(7):e0200418. doi: 10.1371/journal.pone.0200418 (PMC6039016; doi:10.1371/journal.pone.0200418)
Supplement: S7 File — (DOCX) [file pone.0200418.s007.docx]

Monitored feeding form

**COWPEA FORTIFIED PORRIDGE ACCEPTABILITY STUDY**

**OBSERVED FEEDING**

STUDY ID:_________________________ DATE:_____________________________ Day / Month / Year

AGE (mos): _____________________ GENDER: **male / female**

1. What is the child’s mood: **drowsy alert and calm**

**fussy irritable and crying**

1. When was the child last fed (time): **yesterday / this morning / this afternoon**

1. a. Time started feeding b. Time stopped feeding

|___|___| |___|___| |___|___| |___|___|

hr min hr min

1. Amount of food consumed:
2. Based on the following scale, how much does the mother think the child likes the fortified porridge?

[
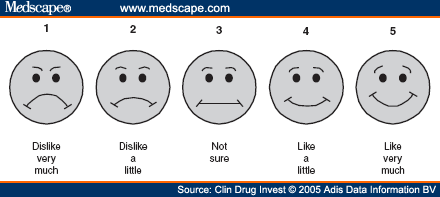
](https://www.google.com/url?sa=i&rct=j&q=&esrc=s&source=images&cd=&ved=0ahUKEwieiJXPtbbMAhWGvxQKHWiBCXUQjRwIBw&url=http://www.medscape.com/viewarticle/504566_2&psig=AFQjCNGdsoLJwwRWX7A0xEGvQXUzooUPjw&ust=1462107547486368)

1. Approximately how much food was wasted or spilled by child (spoonfuls)? **0 / 1 / 2 / 3 / 4 / 5 or more**
2. Did the child need coaxing or support to eat the study food? **Yes / No**
3. Did the child ask for more study food to eat? **Yes / No**
4. General observations ___________________________________________________________________________________

___________________________________________________________________________________
