# Supplementary material for: Effect of cowpea flour processing on the chemical properties and acceptability of a novel cowpea blended maize porridge
Source: PLoS One. 2018 Jul 10;13(7):e0200418. doi: 10.1371/journal.pone.0200418 (PMC6039016; doi:10.1371/journal.pone.0200418)
Supplement: S8 File — (DOCX) [file pone.0200418.s008.docx]

Caregiver acceptability form

**KAFUKUFUKU WA KALANDIRIDWE KA PHALA LOMWE LAIKIDWA UFA WA KHOBWE**

**MOMWE WOYANG’ANIRA MWANA AKONDERA PHALALI**

Nambala ya kafukufuku:_________________________ Tsiku:_____________________________ Tsiku / Mwezi / Chaka

Usinkhu (miyezi): _____________________ **mwamuna / mkazi**

1. Kodi maganizo anu ndi otani pa momwe mwana wanu walikondera phala la khobweli;

[
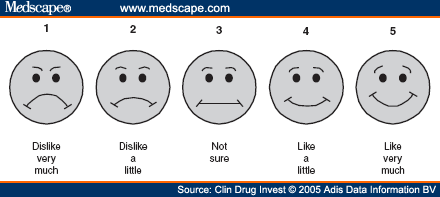
](https://www.google.com/url?sa=i&rct=j&q=&esrc=s&source=images&cd=&ved=0ahUKEwieiJXPtbbMAhWGvxQKHWiBCXUQjRwIBw&url=http://www.medscape.com/viewarticle/504566_2&psig=AFQjCNGdsoLJwwRWX7A0xEGvQXUzooUPjw&ust=1462107547486368)

1. Kodi mutapatsidwa phalali kwanthawi yayitali, mungamamupatse mwana wanu mowirikiza bwanji;

**___ Kawiri pa tsiku**

**___ Kamodzi patsiku**

**___ Kamodzi pasabata**

**___ Kamodzi pa mwezi**

**___ Sindingamamupatse**

1. Ngati simungamamupatse, nchifukwa chiyani? **Ndilovuta kuphika / mwana wanga sangadye / sindikufuna kuyesa chakudya chatsopano / zifukwa zina ______**
2. Kodi mwana wanu wakhalapo ndi vuto lililonse la za umoyo atadya phala la khobweli **Eya / Ayi**

4a. Ngati Eya, Ndi mavuto anji?

**___** Samatha kumeza

**___** Kutsekula mmimba

**___** Kupweteka kwa mmimba

**___** Kulilalila

**___** Zotuluka zapa khungu

**___** Zina __________

1. Ndemanga zina

___________________________________________________________________________________

**__________________________________________________________________________________________**

1. Zomwe ofunsa mafunso waona

**__________________________________________________________________________________________**

**__________________________________________________________________________________________**
