# Supplementary material for: Effect of cowpea flour processing on the chemical properties and acceptability of a novel cowpea blended maize porridge
Source: PLoS One. 2018 Jul 10;13(7):e0200418. doi: 10.1371/journal.pone.0200418 (PMC6039016; doi:10.1371/journal.pone.0200418)
Supplement: S9 File — (DOCX) [file pone.0200418.s009.docx]

Caregiver acceptability form

**COWPEA FORTIFIED PORRIDGE ACCEPTABILITY STUDY**

**CAREGIVER FEEDBACK/ACCEPTABILITY**

STUDY ID:_________________________ DATE:_____________________________ Day / Month / Year

AGE (mos): _____________________ GENDER: **male / female**

1. What is your opinion on the food the child is receiving;

[
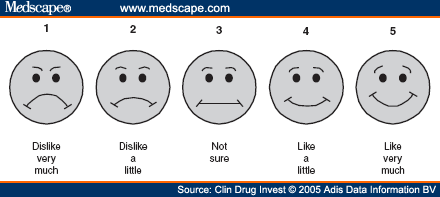
](https://www.google.com/url?sa=i&rct=j&q=&esrc=s&source=images&cd=&ved=0ahUKEwieiJXPtbbMAhWGvxQKHWiBCXUQjRwIBw&url=http://www.medscape.com/viewarticle/504566_2&psig=AFQjCNGdsoLJwwRWX7A0xEGvQXUzooUPjw&ust=1462107547486368)

1. If you were given this food for a long period would you feed it to your child

**___ 2 times per day**

**___ 1 time per day**

**___ 1 time per week**

**___ 1 time per month**

**___ Never**

1. If not, why? **Too hard to make / Child won’t eat it / Worried about feeding new food / Other ______**
2. Has the child had any health problems after eating the food **Yes / No**

4a. If so, what problems?

**___** Couldn’t swallow

**___** Diarrhea

**___** Stomach pains

**___** Crying more

**___** Skin rash

**___** Other __________

1. General comments by the mother

___________________________________________________________________________________

**__________________________________________________________________________________________**

1. General observation by data collector

**__________________________________________________________________________________________**

**__________________________________________________________________________________________**
